# Dynamic Changes in the Levels of Amyloid-β_42_ Species in the Brain and Periphery of APP/PS1 Mice and Their Significance for Alzheimer’s Disease

**DOI:** 10.3389/fnmol.2021.723317

**Published:** 2021-08-27

**Authors:** Liding Zhang, Changwen Yang, Yanqing Li, Shiqi Niu, Xiaohan Liang, Zhihong Zhang, Qingming Luo, Haiming Luo

**Affiliations:** ^1^Britton Chance Center for Biomedical Photonics, Wuhan National Laboratory for Optoelectronics, Huazhong University of Science and Technology, Wuhan, China; ^2^MoE Key Laboratory for Biomedical Photonics, School of Engineering Sciences, Huazhong University of Science and Technology, Wuhan, China; ^3^School of Biomedical Engineering, Hainan University, Haikou, China

**Keywords:** Alzheimer’s disease, blood Aβ_42_, intestinal Aβ, dynamic distribution, ELISA – enzyme-linked immunosorbent assay

## Abstract

Although amyloid-β_42_ (Aβ_42_) has been used as one of the core biomarkers for Alzheimer’s disease (AD) diagnosis, the dynamic changes of its different forms in the brain, blood, and even intestines and its correlation with the progression of AD disease remain obscure. Herein, we screened Aβ_42_-specific preferred antibody pairs 1F12/1F12 and 1F12/2C6 to accurately detect Aβ_42_ types using sandwich ELISA, including total Aβ_42_, Aβ_42_ oligomers (Aβ_42_Os), and Aβ_42_ monomers (Aβ_42_Ms). The levels of Aβ_42_ species in the brain, blood, and intestines of different aged APP/PS1 mice were quantified to study their correlation with AD progression. Total Aβ_42_ levels in the blood were not correlated with AD progression, but Aβ_42_Ms level in the blood of 9-month-old APP/PS1 mice was significantly reduced, and Aβ_42_Os level in the brain was significantly elevated compared to 3-month-old APP/PS1, demonstrating that the levels of Aβ_42_Ms and Aβ_42_Os in the blood and brain were correlated with AD progression. Interestingly, in 9-month-old APP/PS1 mice, the level of Aβ_42_ in the intestine was higher than that in 3-month-old APP/PS1 mice, indicating that the increased level of Aβ_42_ in the gastrointestinal organs may also be related to the progression of AD. Meanwhile, changes in the gut microbiota composition of APP/PS1 mice with age were also observed. Therefore, the increase in Aβ derived from intestinal tissues and changes in microbiome composition can be used as a potential early diagnosis tool for AD, and further used as an indicator of drug intervention to reduce brain amyloid.

## Introduction

Alzheimer’s disease (AD) is an age-related, irreversible form of dementia that affects nearly 50 million people worldwide (Collaborators, 2019). A defining pathological feature of AD is the presence of extracellular deposits of aggregated amyloid-β (Aβ) in the form of senile plaques in specific brain tissues and vascular walls (Murphy and LeVine, 2010), the main components of which are the peptide isoforms Aβ_40_ and Aβ_42_, of which Aβ_42_ predominates in neuritic plaques of AD (Gu and Guo, 2013; Hong and Yaqub, 2019). Autopsy and Aβ positron emission tomography (PET) have indicated that Aβ deposition precedes cognitive decline by a decade or more (Driscoll et al., 2006; Sperling et al., 2011; Oxtoby et al., 2018). Aβ, a 4–4.5 kDa peptide containing 39–42 residues, is produced by sequential proteolytic cleavage of amyloid precursor protein (APP), which is expressed in brain cells and peripheral tissues (such as adrenal gland, kidney, heart, liver, spleen, muscles, and blood vessels) (Roher et al., 2009). Considering that skeletal muscle, accounting for 40% of total body weight (Kim et al., 2016), is only one of many peripheral sources of Aβ, peripheral Aβ may account for a large portion of total Aβ. As observed in human and animal models, brain-derived Aβ peptides can be transported from the brain to peripheral blood (Roberts et al., 2014; Xiang et al., 2015). The communication between the periphery organs and the brain allows for active and dynamic Aβ exchange in distinct reservoirs. The brain accumulation of Aβ aggregates is affected by the levels of Aβ_42_ in the brain and peripheral tissues, but the contribution of peripheral Aβ toward the progression of AD is poorly understood.

Aβ_42_ monomers (Aβ_42_Ms), mainly α-helical and random coil structures, easily aggregate to form various soluble oligomers. The transition of monomers to oligomers triggers the aggregation and pathogenic transformation of Aβ peptides (Nag et al., 2011). A high concentration of Aβ_42_ peptide promotes the formation of its aggregates from soluble monomers into toxic Aβ_42_ oligomers (Aβ_42_Os), and eventually forms extracellular neurotoxic plaques. It has been shown that the brain level of Aβ_42_ increases in the early stage of AD, but decreases with the decline of cognitive ability in the late-stage AD (Naslund et al., 2000). The balance between Aβ production and clearance determines the concentration of Aβ in various reservoirs (Wang et al., 2006). Increasing evidence has implicated that diffusible and soluble Aβ oligomers (Bernstein et al., 2009), rather than insoluble fibrils and small monomers, are the main form of neurotoxicity. These oligomers induce neurotoxic intracellular signaling pathways such as neuronal injury (Wang W. et al., 2019), inflammatory (Salminen et al., 2008), mitochondrial dysfunction, and oxidative stress (Mucke and Selkoe, 2012). The fluctuation of amyloid subtypes in cerebrospinal fluid and blood mainly depends on Aβ_42_Ms and Aβ_42_Os, which are used as core biomarkers to reflect the progression of AD (Benilova et al., 2012). Therefore, dynamic measurement of the levels and profile of Aβ_42_Ms and Aβ_42_Os in the brain and periphery is crucial for studying their physiological metabolism and their substantial correlation with AD progression.

The reliable detection of Aβ_42_Ms and Aβ_42_Os is technically challenging because Aβ_42_Os are transient, heterogeneous, and in dynamic equilibrium with Aβ_42_Ms. In this study, we screened two sequence- and conformation-specific antibodies 1F12 and 2C6, that recognize different epitopes of Aβ_42_. The preferred antibody pairs 1F12/2C6 and 1F12/1F12 were chosen to evaluate the potential significance of various sources and pools of Aβ_42_ on the overall pathology of AD. The correlation between blood Aβ_42_ levels and brain Aβ_42_ levels in APP/PS1 and age-matched C57BL/6J mice at 3 and 9 months was dynamically assessed. Another comprehensive longitudinal study determined the potential effects of intestinal tissue-derived Aβ_42_ on blood and brain Aβ_42_ levels in APP/PS1 mice. We expect that our research will promote the understanding of the role of Aβ_42_Ms and Aβ_42_Os in the progression of AD.

## Materials and Methods

### Chemicals and Materials

The Aβ_3__–__9_, Aβ_13__–__19_, Aβ_18__–__25_, Aβ_29__–__36_, Aβ_36__–__4__2_, Aβ_40_, and Aβ_42_ were custom-synthesized as lyophilized powders by Royo Biotech Co., Ltd (Shanghai, China) with a purity of >95%. Anti-Aβ (6E10) antibody was obtained from Invitrogen. The goat anti-mouse IgG (H + L), protein A resin, and protein L resin were ordered from GenScript (Nanjing, China). The mouse monoclonal antibody isotyping kit was purchased from Southern Biotech (Birmingham, AL, United States). The Pierce streptavidin-coupled poly-HRP and protein marker were ordered from Thermo Scientific (Massachusetts, United States). Thioflavin S, BSA, Freund’s complete adjuvant, and Freund’s incomplete adjuvant were obtained from Sigma-Aldrich. Cy3-NHS ester and Biotin-PEG4-NHS ester were provided by Lumiprobe (Hannover, Germany). All other chemicals were purchased from commercial suppliers and used as received.

### Oligomeric and Monomeric Aβ Preparations

The Aβ_40_ monomers (Aβ_40_Ms) and Aβ_42_Ms were obtained by dissolving lyophilized Aβ_40_, Aβ_42_ peptides in 1,1,1,3,3,3-hexafluoroisopropanol (HFIP), followed by incubation overnight at room temperature. HFIP was evaporated with nitrogen gas to form a film, and the Aβ was redissolved in dimethyl sulfoxide. The prepared Aβ_40_, Aβ_42_ monomers solution (50 μM) was stored at −20°C as stock solution. The Aβ_40_ oligomers (Aβ_40_Os) and Aβ_42_ oligomers (Aβ_42_Os) were obtained from 24 h incubation of 50 μM Aβ_40_ and Aβ_42_ monomer solutions at 37°C in the dark, respectively.

### Cryo-Transmission Electron Microscopy

The patterns of the prepared Aβ_42_Ms and Aβ_42_Os were confirmed by cryo-transmission electron microscopy (Cryo-TEM). In brief, 5 μL of each sample was deposited on a copper grid, and the excess liquid was removed using a filter paper, leaving a thin film of the solution on the grid. The Tecnai G20 transmission electron microscope (FEI Ltd., United States) was used to characterize the morphology of the above-mentioned samples.

### Generation and Purification of 1F12 and 2C6

The antigen was prepared using lyophilized synthetic Aβ_42_ peptide dissolved in 10 mM NaOH and diluted with PBS to a final concentration of 1 mg/mL. In the first immunization, BALB/c mice were immunized with 50 μg Aβ_42_ peptide mixed with Freund’s complete adjuvant. In the second and third immunizations, Aβ_42_ peptides and Freund’s incomplete adjuvant mixtures were used (Zhang et al., 2018). After the third immunizations, 50 μg Aβ_42_ peptide was used for booster immunization. Three days later, spleen cells were collected from the immunized mice and fused with SP2/0 cells via PEG at 37°C (Wang X. et al., 2019). The fused cells were maintained in HAT medium for 7 days and then cultured in HT medium. The positive hybridomas in each plate were screened by the limited dilution method (Kohler and Milstein, 1975). Three positive hybridoma cell lines were screened from the initial positive wells and cultured to prepare ascites. The immunoglobulins of 1F12, 2C6, and 2E2 were purified using protein A resin, according to the manufacturer’s instructions.

### Dot Blot Assay

One microgram of each Aβ_42_ truncated peptide including Aβ_3__–__9_, Aβ_13__–__19_, Aβ_18__–__25_, Aβ_29__–__36_, and Aβ_36__–__42_ was pipetted onto a PVDF membrane activated by methanol. After the peptide samples were deposited, the PVDF membrane was air-dried and blocked in 5% skimmed milk in phosphate-buffered saline (PBS) with Tween-20 detergent (PBS-T) at 37°C for 1 h. The membranes were then incubated with mAb 1F12 (1:1000) or 2C6 (1:1000) at 37°C for 2 h. After washing three times in PBS-T, the membrane was incubated with the secondary antibody HRP-conjugated goat anti-mouse IgG (H + L) at 37°C for 2 h, and the immunological signals were detected with ECL-substrate (Vazyme, China) using Tanon 5200 Muiti (Shanghai, China).

### Biotinylated 1F12 or 2C6 Antibody Preparation

The biotinylated antibodies were generated based on the reaction of amino groups of 1F12 or 2C6 antibody with biotin *N*-hydroxysuccinimide (NHS) ester. Briefly, 10 nmol of 1F12 or 2C6 antibody was dissolved in PBS, and the solution pH was adjusted to 8.5 with Na_2_CO_3_ (0.1 M), followed by a reaction with 20 nmol biotin-PEG4-NHS esters in DMSO at room temperature for 2 h. The biotin-modified 1F12 or 2C6 antibody was purified using size exclusion PD-10 columns with PBS as the mobile phase. Indirect ELISA determined the activities and titers of biotinylated 1F12 or 2C6. The indirect ELISA assay was performed using serially diluted biotin-labeled antibodies (from 1:100 to 1:409,600) instead of the primary antibody. After incubation with the Pierce streptavidin-coupled poly-HRP, the immunoreaction was visualized with a soluble TMB substrate solution.

### Measurement of the Titer and Binding Affinity of 1F12 or 2C6 Antibody

The 96-well plates were coated with 0.5 μg/well of Aβ_42_ and blocked with 5% skimmed milk dissolved in a PBS-T buffer for 2 h at room temperature. Subsequently, series of diluted 1F12 or 2C6 ranging from 1:100 to 1:409,600 were added into each well for 2-h incubation at 22°C. The subsequent steps were performed as described in the ELISA section above. The binding affinities of 1F12 or 2C6 with Aβ_42_ species were determined using serially diluted preparations of 1F12 or 2C6 with serials of concentrations (from 10 μg/mL to 10 ng/mL) instead of different dilutions (from 1:100 to 1:409,600). After incubation with the HRP-conjugated goat anti-mouse IgG (H + L), the immunoreaction was visualized with a soluble TMB substrate solution.

### Screening of Preferred Antibody Pairs for Total Aβ_42_ and Aβ_42_Os Sandwich ELISA

Preferred antibody pairs of total Aβ_42_ and Aβ_42_Os sandwich ELISA were screened from the combination of different antibody pairs. The screening process of antibody pairs for total Aβ_42_ was as follows: 96-well plate was coated with 1 μg/well of 1F12 or 2C6 as the capture antibody for 2 h at room temperature, and then blocked with 5% skimmed milk. Five ng total Aβ_42_ containing Aβ_42_Ms and Aβ_42_Os or Aβ_42_Os was loaded into each well and incubated 2 h at room temperature, followed by the addition of biotinylated 2C6 or 1F12 as the detection antibody. Finally, streptavidin-coupled poly-HRP was used to visualize the immunoreaction of each well.

### Sandwich ELISA for the Detection of Total Aβ_42_ and Aβ_42_Os

The 96-well plates were coated with 1 μg/well of 1F12 in the citrate-buffered saline buffer for 2 h, and then blocked with 5% skimmed milk dissolved in PBS-T for 2 h at room temperature. A standard series of synthetic Aβ peptides (Aβ_40_Ms, Aβ_42_Ms, Aβ_40_Os, and Aβ_42_Os) and the biological samples (mouse blood and organ homogenization) to be analyzed were added to the plates in triplicates and incubated for 40 min at room temperature. The plate was washed three times with PBS-T and incubated with biotinylated mAb 2C6 for total Aβ_42_ detection or biotinylated mAb 1F12 for Aβ_42_Os detection, followed by incubation with streptavidin-coupled poly-HRP for 1 h at room temperature. The immunoreaction in each well was detected using TMB substrate solution.

### Quantification of Aβ Peptides in Blood, Brain, and Intestinal Tissue

All procedures involving animal studies were reviewed and approved by the Institutional Animal Care and Use Committee of Huazhong University of Science and Technology. Herein, 3-month-old APP/PS1 mice (six mice for blood collection including three female and three male mice, and four mice for tissue collection, including two female and two male mice), 9-month-old APP/PS1 mice (six mice for blood collection including three female and three male mice, *n* = 4 for tissue collection, including two female and two male mice), and age-matched non-transgenic C57BL/6J mice (six mice for blood collection, including three female and three male mice, and four mice for tissues collection, including two female and two male mice) were anesthetized with 0.4 mL Avertin (25 mg/mL) and intracardially perfused with 0.9% saline solution. The mouse blood was collected, and tissues were organically extracted with antigen extraction tris-buffered saline (TBS, 20 mM Tris and 137 mM NaCl, pH 7.6) at a ratio of 1:50 (w/v) using a tissue grinder. The TBS included a complete protease inhibitor cocktail (Roche). The supernatant of homogenates was centrifuged at 10,000 *g* for 30 min in Type 60 Ti fixed angle rotor at 4°C to obtain TBS-soluble proteins. The collected mouse blood, prepared brain, and intestinal tissue samples were used for Aβ_42_ quantification by the above-prepared sandwich ELISA.

### Immunoprecipitation and Western Blotting

The homogenates of organ tissues (brain, stomach, duodenum, jejunum, ileum, cecum, colon, and intestinal lysates) or blood were incubated for 30 min at room temperature with 40 μg/mL of 1F12 antibody conjugated on the protein A/G magnetic bead, according to the manufacturer’s instructions. The immunoprecipitated proteins were eluted with 0.1 M glycine (pH 3.0) and immediately neutralized to pH 7.4 using a neutralization buffer (1 M Tris-HCl, pH 8.5). The samples were then denatured in loading buffer (Boster Biotech, United States) and boiled for 10 min. Following this, the proteins were run on a 12% reduced tris-tricine SDS-polyacrylamide gel via SDS-PAGE. Proteins were transferred onto a polyvinylidene fluoride membrane at 160 mA for 1 h, at 4°C. Membranes were blocked with 5% skimmed milk dissolved in 1 × PBS-T and incubated with 1F12 or 2C6 for 2 h at 37°C. Subsequently, the membranes were washed in PBS-T followed by 1 h incubation with secondary antibody HRP-conjugated goat anti-mouse IgG (H + L), and the immunological signals were detected using the ECL-substrate (Vazyme, China) on Tanon 5200 Muiti (Shanghai, China).

### Immunofluorescence Assays of Tissues

The mice were anesthetized with 0.4 mL Avertin (25 mg/mL), and cardiac perfusion was performed with 4% paraformaldehyde (PFA) for 30 min. The tissues (brain, duodenum, jejunum, ileum, cecum, and colon) from mice were collected, fixed in 4% PFA, and dehydrated in sucrose solution. After embedding in OCT compound (Tissue-Tek; Sakura Finetek, Netherlands, United States), 15 μm coronal frozen sections of tissue samples were serially cut on a Leica CM3050 S cryostat. Each slice was mounted on glass slides and permeabilized with 0.2% Triton X-100 overnight at 4°C. The slice was blocked with 3% BSA for 2 h at room temperature and incubated with thioflavin S or commercially available anti-Aβ antibody 6E10, Cy3-conjugated 1F12 or 2C6 overnight at 4°C. All slides were washed five times with PBS and stained with thioflavin S. The Zeiss LSM710 microscope was used to image slides with red, green, and blue fluorescence filters.

### Sequencing Data and Statistical Analyses

Sequencing data from an independent study of the APP/PS1 mice gut microbiota was obtained. Raw 16S sequences were downloaded from SRA accession PRJNA543965 and preprocessed using the ‘dada2’ R package. Reads were truncated at 150 bp, then a maximum expected error rate threshold of 1 was imposed. Taxonomy was assigned using the Silva v138 rRNA database using the default classify algorithm of dada2 (v1.16.0).

Downstream analysis was performed by several R packages including ‘phyloseq,’ ‘MicrobiotaProcess,’ ‘DESeq2,’ and ‘ggplot2.’ Beta diversity was measured by principal coordinate analysis (PCoA) based on weighted UniFrac phylogenetic distance. The significantly differed genus between groups was determined using ‘DESeq2,’ and their significance of difference was assessed by Wilcoxon rank-sum test. The significance cut-off was set at ^∗^*p* < 0.05.

The data, except for sequencing data, are presented as means ± SEM. One-way or two-way analysis of variance (ANOVA) was used for multiple group comparisons. Statistical significance is represented in the figure by ^∗^*p* < 0.05, ^∗∗^*p* < 0.01, ^∗∗∗^*p* < 0.001, ^****^p < 0.0001, and n.s. (indicating no significance). All statistical analyses were performed with GraphPad Prism7.0 software.

## Results

### Screening and Identification of Aβ_42_ Sequence- and Conformation-Specific Antibodies

**FIGURE 1:**
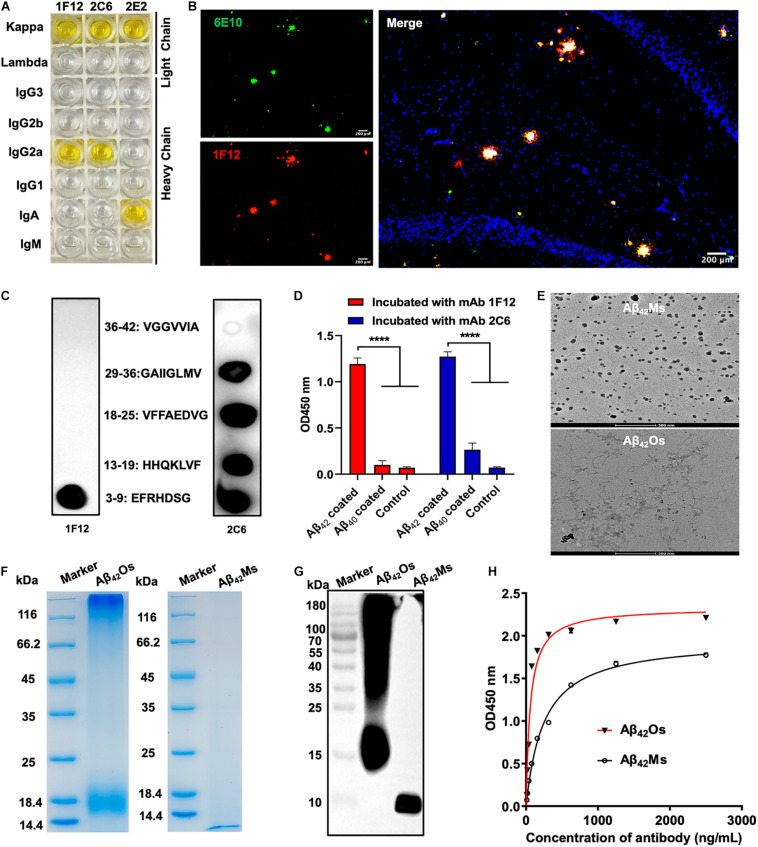
Characterization of sequence and conformation-specific antibodies for Aβ_42_Ms and Aβ_42_Os. **(A)** The isotypes of the prepared monoclonal antibodies. **(B)** Confocal fluorescence images of murine APP/PS1 brain sections using Cy3-labeled anti-Aβ_42_ monoclonal antibody 1F12 and commercially available anti-Aβ antibody 6E10. (Scale bar: 200 μm). **(C)** Linear epitopes of Aβ_42_ recognized by 1F12 and 2C6. **(D)** The binding affinities of 1F12 and 2C6 to Aβ_42_ and Aβ_40_ were determined by indirect ELISA. **(E)** The morphologies of Aβ_42_Ms and Aβ_42_Os were analyzed by cryo-transmission electron microscopy in parallel. (Scale bar: 500 nm). The purity and molecular weight of freshly prepared Aβ_42_Ms and Aβ_42_Os were determined by 12% reduced SDS-PAGE using Coomassie blue staining **(F)** and Western blotting with 1F12 **(G)**. **(H)** The *K*_d_ value of 1F12-binding Aβ_42_Ms and Aβ_42_Os was detected with indirect ELISA. Data are presented as means ± SEM. One-way analysis of variance (ANOVA) was used for multigroup comparisons. Statistical significance is indicated in the figures by ****p < 0.0001.

BALB/c mice were immunized with human Aβ_42_ peptide preparations, and a pool of approximately 1,500 clones was generated via hybridoma technology. In addition, 75 of these clones reacted with Aβ monomers and oligomers in ELISA (Supplementary Figure 1A). Then three positive hybridoma clones 1F12, 2C6, and 2E2 were selected, and their immunoglobulin isotypes were determined. The results showed that the isotype of 1F12 and 2C6 was IgG2a, and the isotype of 2E2 was IgA, and the light chains of these three monoclonal antibodies (mAbs) all belonged to the kappa chain (Figure 1A). Reduced SDS-PAGE identified the purity and molecular weight of these three mAbs (Supplementary Figure 1B). Therefore, 1F12 and 2C6 were chosen to characterize their reactivity by Western blotting brain natural Aβ_42_ and immunostaining brain slices of APP/PS1 mice. The results displayed strong bands of Aβ_42_ peptides recognized by 1F12 and 2C6, comparable to those obtained with 6E10 (Supplementary Figure 1C). Immunofluorescence imaging (using thioflavin S or commercially available anti-Aβ antibody 6E10) showed that Aβ plaques were colocalized with Cy3-labeled 1F12 (Figure 1B and Supplementary Figure 2A) or 2C6 (Supplementary Figures 1D, 2B). The titer of mAbs was determined by indirect ELISA and calculated as 1:204800 for 1F12, 1:102400 for 2C6, 1:51200 for 2E2 (Supplementary Figure 1E).

To identify the linear fragments (epitopes) of Aβ_42_ recognized by 1F12 and 2C6, we performed epitope mapping experiments using a series of peptides starting from +3 to +42 in the Aβ_42_ sequence. Based on a series of dot-blot analyses, we observed that 1F12 displayed a linear epitope, amino acids 3–9 located in the N-terminal region of the Aβ_42_ peptide (Figure 1C, left). In contrast, 2C6 exhibited discrete epitopes, including four distinct Aβ_42_ fragments (Figure 1C, right). To confirm the binding efficacy of 1F12 and 2C6 to Aβ_42_ and Aβ_40_ peptides, indirect ELISAs were performed on separate Aβ_42_ and Aβ_40_ coated plates. The 1F12 and 2C6 detection signals of Aβ_42_ were significantly higher than those of Aβ_40_ (Figure 1D). Taken together, 1F12 and 2C6 were Aβ_42_ sequence-specific antibodies, and both showed a preference for the conformational epitope presented by Aβ_42_ rather than Aβ_40_.

### Binding Affinities and Selectivity of 1F12 and 2C6 for Different Aβ_42_ Species

We further evaluated the binding affinity of 1F12 and 2C6 to Aβ_42_Ms and Aβ_42_Os with different conformations. Cyo-TEM confirmed the morphology of the prepared Aβ42Ms and Aβ42Os. The results showed that the morphology of Aβ_42_Ms was α-helical and random coil structures (Figure 1E, up), while Aβ_42_Os formed a β-sheet and typical fibril three-dimensional structures (Figure 1E, down). 12% reduced SDS-PAGE gel confirmed the purity and molecular weight of the prepared Aβ_42_Ms and Aβ_42_Os (Figure 1F), and their components were verified by Western blotting (Figure 1G and Supplementary Figure 1F). Indirect ELISA results showed that the *K*_d_ values of 1F12 bound to Aβ_42_ species were 1.66 ± 0.09 nM for Aβ_42_Ms and 0.38 ± 0.04 nM for Aβ_42_Os (Figure 1H), while the *K*_d_ values of 2C6 were 3.59 ± 0.27 nM for Aβ_42_Ms and 0.61 ± 0.03 nM for Aβ_42_Os (Supplementary Figure 1G). Taken together, 1F12 and 2C6 were Aβ_42_ sequence- and conformation-specific antibodies and could bind Aβ_42_ species with different conformations.

### Preferred Antibody Pairs for Specific Detection of Total Aβ_42_ and Aβ_42_Os

**FIGURE 2:**
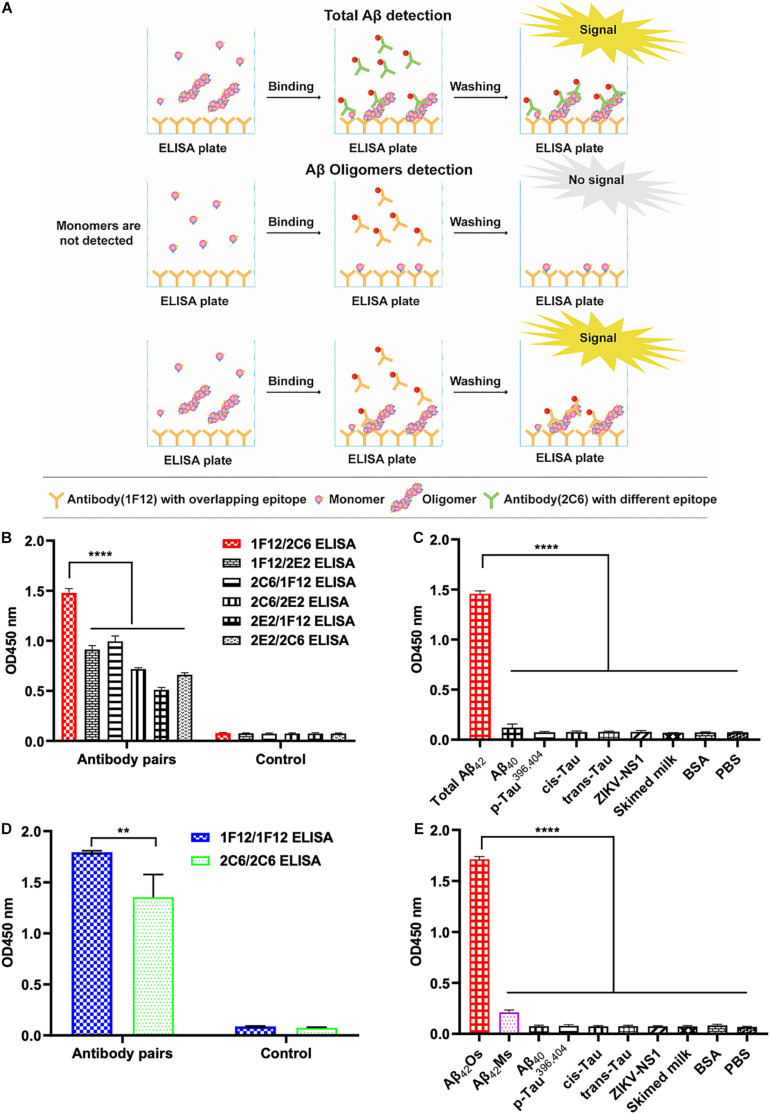
Screening of preferred antibody pairs 1F12/2C6 and 1F12/1F12 against Aβ_42_Ms and Aβ_42_Os. **(A)** Schematic representation of 1F12/2C6, 1F12/1F12 ELISA to detect total Aβ_42_, Aβ_42_Os, respectively. **(B)** Preferred antibody pairs were screened for the detection of total Aβ_42_. **(C)** The specificity assay of 1F12/2C6 ELISA for total Aβ_42_ detection. **(D)** Preferred antibody pairs were screened for the detection of Aβ_42_Os. **(E)** The specificity assay of 1F12/1F12 ELISA for Aβ_42_Os detection. Data are presented as means ± SEM. Two-way analysis of variance (ANOVA) was used for **(B,D)** and One-way analysis of variance (ANOVA) was used for **(C,E)**. Statistical significance is indicated in the figures by **p < 0.01 and ****p < 0.0001.

The screening of capture and detecting antibodies for sandwich ELISA is a prerequisite for developing techniques to detect and quantify Aβ_42_Ms and Aβ_42_Os. Both 1F12 and 2C6 were biotinylated, and indirect ELISA showed that their bioactivities and titers were high (Supplementary Figures 1H,I). To achieve the specific detection of total Aβ_42_ and Aβ_42_Os, a combination of different antibody pairs (1F12/2C6, 1F12/2E2, 2C6/1F12, 2C6/2E2, 2E2/1F12, 2E2/2C6) was screened for preferred antibody pairs by comparing their susceptibility to total Aβ_42_ and Aβ_42_Os in sandwich ELISA (Figure 2A). The capture/detection antibody pair 1F12/2C6 had the highest detection signal and specificity for total Aβ_42_ among the six antibody pairs in ELISA (Figures 2B,C), while the 1F12/1F12 antibody pair was significantly more effective and specificity in detecting Aβ_42_Os (Figures 2D,E).

### Dynamic Monitoring of Changes in Total Aβ_42_, Aβ_42_Os, and Aβ_42_Ms in Blood and Brain

**FIGURE 3:**
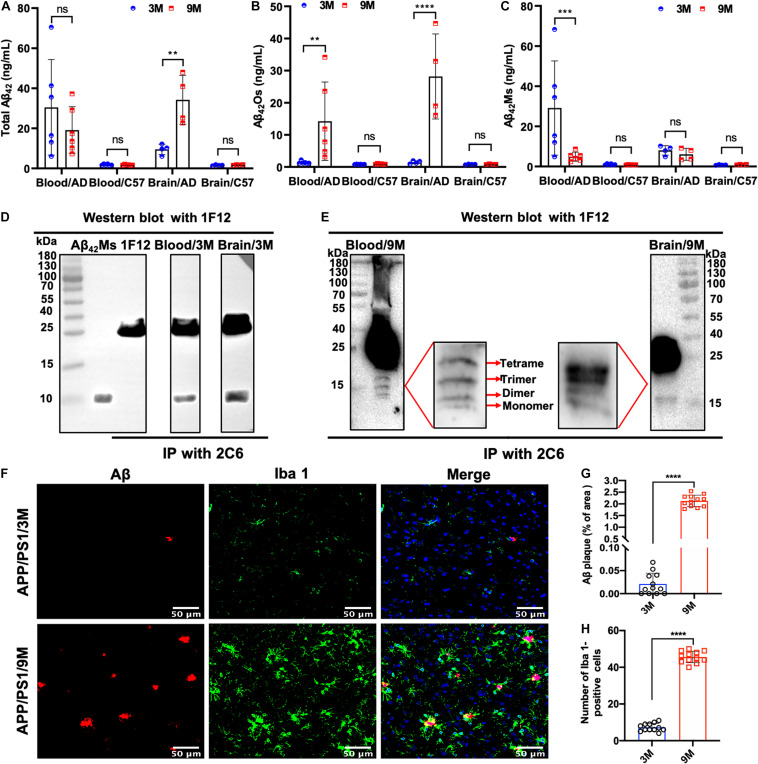
Dynamic detection of the changes of Aβ_42_Ms and Aβ_42_Os in blood and brain of APP/PS1 mice by 1F12/2C6 and 1F12/1F12 ELISA. The level of total Aβ_42_ **(A)**, Aβ_42_Os **(B)**, and Aβ_42_Ms **(C)** in the blood (*n* = 6) and brain (*n* = 4) of 3- and 9-month-old APP/PS1 and C57BL/6J mice. Total Aβ_42_ in the blood and brain of 3 **(D)** or 9-month-old **(E)** APP/PS1 mice were enriched with immunoprecipitation (2C6) and visualized via Western blotting (1F12). **(F)** Confocal fluorescence images of Aβ plaque and Iba 1-positive cells staining in 3 or 9-month-old APP/PS1 mice. Aβ plaque **(G)** and Iba 1-positive **(H)** areas in the brains of 3- or 9-month-old APP/PS1 mice. Data are presented as means ± SEM. Two-way analysis of variance (ANOVA) was used in **(A–C)** for multigroup comparisons. Unpaired t-test was used in **(G,H)** for two-group comparisons. Statistical significance is indicated in the figures by **p < 0.01, ***p < 0.001, ****p < 0.0001, and n.s. (indicating no significance).

To dynamically monitor the changes of total Aβ_42_, Aβ_42_Os, and Aβ_42_Ms in the brain and periphery blood, 1F12/2C6 and 1F12/12 ELISAs were performed to quantify Aβ_42_ in peripheral blood and brain extracts of 3 and 9-month-old APP/PS1 mice. The results showed that regardless of age (at 3- or 9-month-old), the total Aβ_42_ in blood and brain extracts of APP/PS1 mice was significantly higher than that in the tissue extracts of C57BL/6J (Figure 3A). 1F12/2C6 ELISA showed that compared with the 3-month-old APP/PS1, the total Aβ_42_ levels in the blood of the 9-month-old APP/PS1 did not change significantly (*p* = 0.1787), but the Aβ_42_ content in the brain tissue was significantly increased (*p* = 0.0062, Figure 3A). In comparison, 1F12/1F12 ELISA showed that the Aβ_42_Os level in the blood (*p* = 0.005) and brain (*p* < 0.0001) of APP/PS1 at 9 months old were significantly higher than that of APP/PS1 mice at 3 months old (Figure 3B). The amount of Aβ_42_Ms was calculated by subtracting the total Aβ_42_ from Aβ_42_Os. The level of Aβ_42_Ms in the blood of APP/PS1 mice at 9 months old was significantly lower than that of APP/PS1 mice at 3 months old (*p* = 0.0004, Figure 3C). The levels of total Aβ_42_, Aβ_42_Os, and Aβ_42_Ms in C57BL/6J mice between 3 and 9 months old did not change significantly (Figures 3A–C). To further validate the 1F12/2C6 and 1F12/1F12 ELISA results, an immunoprecipitation (IP) assay was performed using blood and brain extracts of 3- and 9-month-old APP/PS1 mice. As shown in Figure 3D, a clear single band was observed in the blood and brain extracts of 3-month-old APP/PS1, and its molecular weight was similar to Aβ_42_Ms. The results were consistent with the ELISA results as above-mentioned, confirming that the level of Aβ_42_Ms at the early stage of AD (3-month-old APP/PS1) was high and the level of Aβ_42_Os in the late stage of AD (9-month-old APP/PS1) were elevated. Western blotting analysis of immunoprecipitated proteins in blood and brain extracts of 9-month-old APP/PS1 mice revealed a prominent Aβ_42_Os band with a molecular weight of more than 5 kDa and several clear bands of monomers to tetramers (Figure 3E). Taken together, Aβ_42_ mainly existed as a monomer in the blood at the early stage of AD (e.g., 3-month-old APP/PS1 mice with less Aβ plaque load and Iba 1-positive cells staining, Figures 3F–H), whereas it appears as oligomers in blood and brain extracts at the late state (e.g., 9-month-old APP/PS1 mice with more Aβ plaque load and Iba 1-positive cells staining, Figures 3F–H).

### Correlation of Total Aβ_42_, Aβ_42_Os, and Aβ_42_Ms Level in the Gastrointestinal System With AD Progression

**FIGURE 4:**
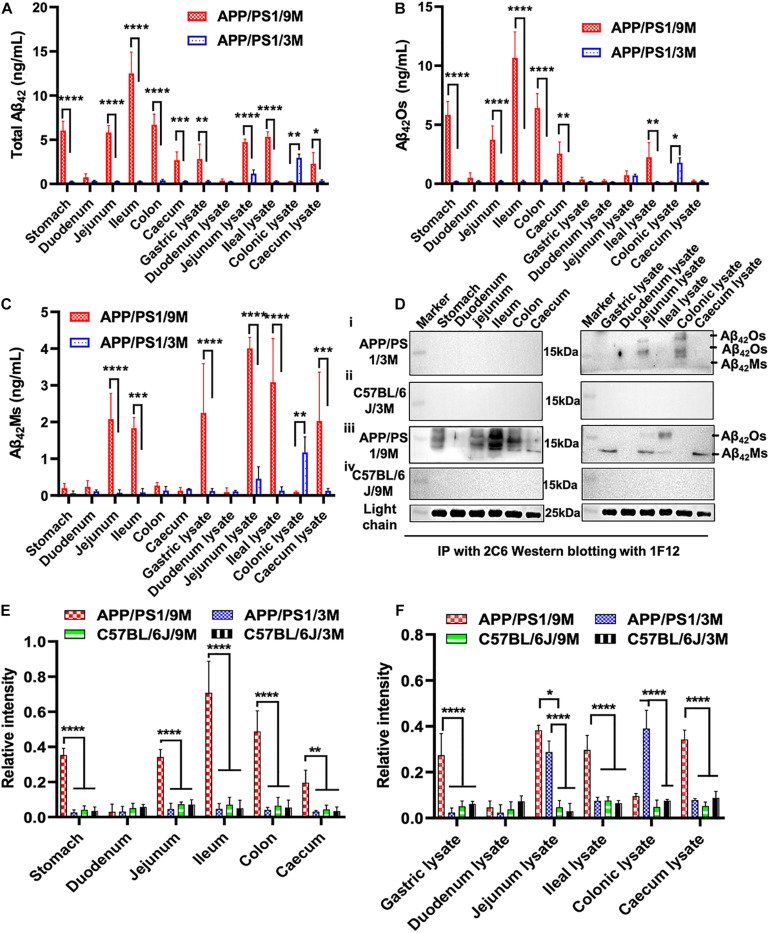
Dynamic changes in the levels of Aβ_42_Ms and Aβ_42_Os in the intestines were detected and quantified via 1F12/2C6 and 1F12/1F12 ELISA. The level of total Aβ_42_, Aβ_42_Os, and Aβ_42_Ms in the stomach, duodenum, jejunum, ileum, colon, and cecum and their lysates from 3-month-old APP/PS1 (*n* = 4; **A–C**), 9-month-old APP/PS1 (*n* = 4; **A–C**). **(D)** Representative IP-Western blotting data to analyze the distribution of Aβ_42_ in the stomach, duodenum, jejunum, ileum, colon, and cecum and their lysates from 3-month-old APP/PS1 (i), 9-month-old APP/PS1 (iii), and C57BL/6J mice (ii,iv) at the same ages. **(E,F)** The Western blotting results for Aβ_42_ in APP/PS1 or C57BL/6J mice were quantified. Data are presented as means ± SEM. Two-way analysis of variance (ANOVA) was used for multigroup comparisons. Statistical significance is indicated in the figures by *p < 0.05, **p < 0.01, ***p < 0.001, and ****p < 0.0001.

Apart from blood and brain, we also paid attention to the distribution of Aβ_42_ in the intestinal system of APP/PS1 mice, such as duodenum, jejunum, ileum, colon, cecum, and their lysates. Several research groups have reported that AD may begin in the intestine and is closely related to the imbalance of intestinal flora (Hu et al., 2016; Harach et al., 2017; Jiang et al., 2017; Mancuso and Santangelo, 2018). To detect the distribution of total Aβ_42_, Aβ_42_Ms, and Aβ_42_Os in the intestinal systems and explore its potential correlation with the pathogenesis of AD, organs extracted from different parts of intestines (duodenum, jejunum, ileum, colon, and cecum) and their lysates from 3- and 9-month-old APP/PS1 mice were collected for 1F12/2C6 and 1F12/1F12 ELISA. No Aβ_42_ was detected in the different parts of intestines in 3-month-old APP/PS1 mice, but a certain amount of Aβ_42_ was observed in jejunum lysate and colonic lysate (Figure 4A). Further analysis revealed Aβ_42_ subtype in the jejunum and colon lysates. It was found that Aβ_42_Os were observed in both jejunum and colonic lysate, with higher levels of Aβ_42_Os in colon lysates (Figure 4B). But for Aβ_42_Ms, a weak signal was only observed in the colonic lysate (Figure 4C). However, for 9-month-old APP/PS1 mice, total Aβ_42_ signals were clearly observed in all gastrointestinal organs and their lysates, except for duodenum, duodenum lysates, and colonic lysates (Figure 4A). In a detailed analysis, we found that Aβ_42_ mainly existed in the form of oligomers and was detected in all gastrointestinal organs except for the duodenum (Figure 4B). For the lysates, the result was opposite to the Aβ_42_Ms level, and only two oligomers were detected with weak signals in the jejunum and ileum lysates (Figure 4C). For 3 or 9-month-old C57BL/6J mice, no significant differences were observed in the gastrointestinal organs and their lysates (Supplementary Figures 3A,D). Interestingly, the monomeric (Supplementary Figures 3C,F) or oligomeric (Supplementary Figures 3B,E) forms of Aβ_42_ levels in APP/PS1 mice were significantly higher than those in the C57BL/6J at 3 or 9 months old. Altogether, the ELISA results confirmed that both Aβ_42_Ms and Aβ_42_Os exist in the gastrointestinal system, and their distributions are different in APP/PS1 mice, but their levels increase with age.

Meanwhile, IP-Western blotting further confirmed the presence of Aβ_42_ in the intestine through 1F12/2C6 and 1F12/1F12 ELISA. The Aβ_42_Os signal was only observed in jejunal lysates and colon lysates of 3-month-old APP/PS1 mice (Figure 4D, i). In comparison, no Aβ_42_ species were detected in 3-month-old C57BL/6J mice (Figure 4D, ii). Compared with 3-month-old APP/PS1 mice, the distribution of Aβ_42_Os and Aβ_42_Ms in 9-month-old APP/PS1 mice was quite different. In 9-month-old APP/PS1 mice, obvious Aβ_42_Os bands were observed in the stomach, jejunum, ileum, colon, cecum, jejunum lysate, and ileum lysate, while Aβ_42_Ms levels were detected in low in the jejunum, ileum, and in high in the lysates of stomach, jejunum, and cecum (Figure 4D, iii). However, in age-matched C57BL/6J mice, no bands of Aβ_42_Ms or/and Aβ_42_Os were observed (Figure 4D, iv). The light chain of 1F12 was used as an internal reference to ensure that the same amount of immunomagnetic beads was added to each sample. The quantitative data of Western blotting (Figures 4E,F) were consistent with the 1F12/2C6 and 1F12/1F12 ELISA results (Figures 4A–C and Supplementary Figures 3A–F), indicating that both the levels of Aβ_42_Ms and Aβ_42_Os and their distributions in the gastrointestinal system are correlated with AD progression.

**FIGURE 5:**
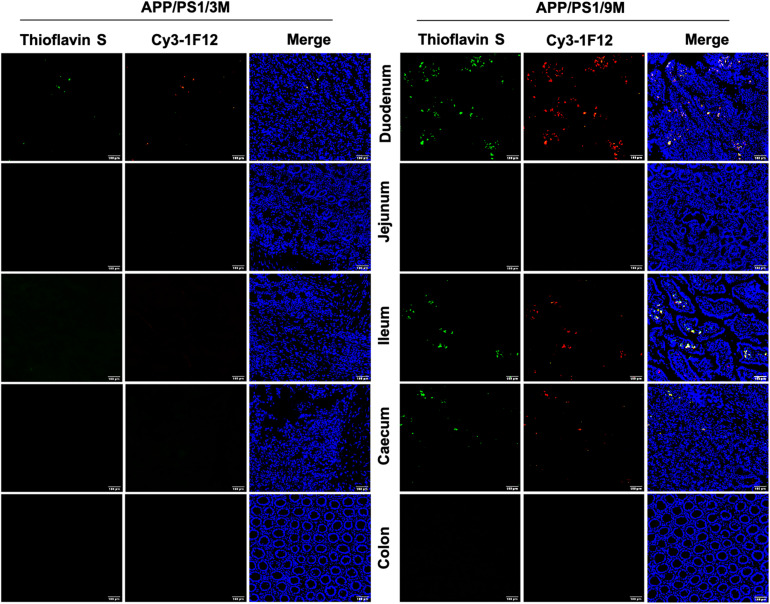
Immunofluorescence assay for insoluble Aβ plaques in different parts of the intestines of APP/PS1 mice. The levels of insoluble Aβ plaques in the duodenum, jejunum, ileum, cecum, and colon of 3-month-old **(left)** and 9-month-old **(right)** APP/PS1 mice were evaluated by double-staining with thioflavin S and Cy3-IF12 (Scale bar: 100 μm).

Based on the results of IP-Western blotting and sandwich ELISA, we further investigated the accumulation of insoluble Aβ plaques in the intestine because Aβ_42_Ms and Aβ_42_Os were the core components of amyloid plaques. The immunofluorescence assay (IFA) showed that the weak fluorescent signals of thioflavin S and Cy3-1F12 were colocalized in the duodenum of 3-month-old mice (Figure 5, left). However, in 9-month-old APP/PS1 mice, obvious fluorescent colocalization signals of thioflavin S and Cy3-1F12 were observed in the duodenum, ileum, and cecum, which of their fluorescence signals were significantly stronger than that of 3-month-old APP/PS1 mice (Figure 5, right). In comparison, in 3- or 9-month-old C57BL/6J mice, no fluorescent signals of thioflavin S and Cy3-1F12 were observed (Supplementary Figure 3 and Figure 4). Overall, the results of sandwich ELISA (Figures 4A–C), IP-Western blotting (Figure 4D), and IFA (Figure 5) convincingly demonstrated that Aβ exists in the intestine of APP/PS1 mice, and their levels correlate to AD progression.

### Dynamic Changes in Microbiome Composition of APP/PS1 Mice With Age

**FIGURE 6:**
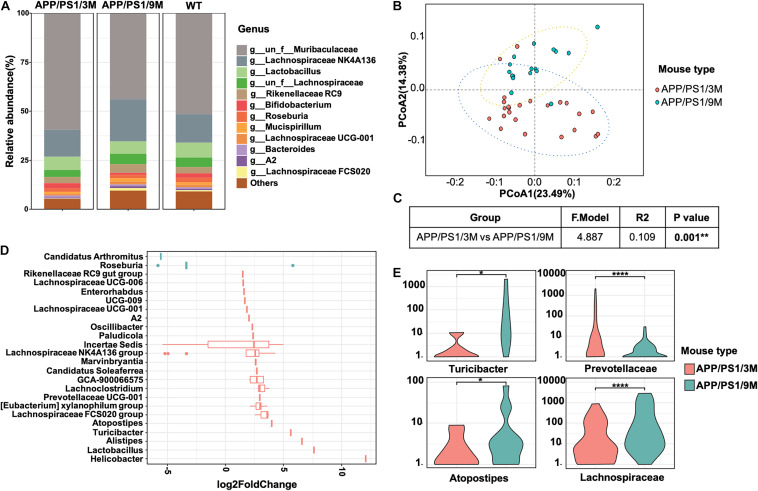
Comparison of microbiome composition of APP/PS1 mice at 3 and 9 months old. **(A)** The relative abundance of top microorganisms in the feces of 3 and 9-month-old APP/PS1 mice and age-matched C57/BL/6 mice. **(B)** Principal coordinate analysis (PCoA) based on weighted UniFrac phylogenetic distance to compare 3 and 9-month-old APP/PS1 mice. **(C)** Comparison of beta diversity between 3 and 9-month-old APP/PS1 mice based on weighted UniFrac phylogenetic distance. The data was calculated using permutational multivariate analysis of variance (PERMANOVA) by R package ‘vegan.’ **p < 0.01. **(D)** Significant difference in the bacterial genus with relative abundance ≥ 0.01% between 3 and 9-month-old APP/PS1 mice. **(E)** The main genus of APP/PS1 mice with significant differences at 3 and 9 months. Comparisons of the relative abundance of gut microbiota between groups at the genus level were performed using two-tailed Wilcoxon rank-sum tests. Statistical significance is indicated in the figures by *p < 0.05, **p < 0.01, and ****p < 0.0001.

To investigate the changes in microbiome composition of APP/PS1 mice with age, we compared relative microbial abundance at genus levels among 3-month-old APP/PS1 mice, 9-month-old APP/PS1 mice, and age-matched C57BL/6J mice. Among all three groups, the abundance of Lachnospiraceae fluctuated with age (Figure 6A). To further characterize the microbiome composition, a beta diversity analysis was performed based on PCoA and weighted UniFrac distance in 3 and 9-month-old APP/PS1 mice (Figure 6B). The PERMANOVA results revealed that the microbiome structure was reshaped during the development of AD (Figure 6C). Then, representative bacterial taxa that differed significantly across two APP/PS1 groups were determined by ‘DESeq2’ R package based on the negative binomial distribution. Taxa were sorted by fold change between the two groups at the genus level (Figure 6D). In addition, the content of Turicibacter, Atopostipes, and Lachnospiraceae in 9-month-old APP/PS1 mice was significantly higher than that in 3-month-old mice (Figure 6E). Meanwhile, the Prevotellaceae was less abundant in aged APP/PS1 mice (Figure 6E). Collectively, these results illustrated that in the early stages of AD, the composition of the gut microbiota shows a significant difference.

## Discussion

In this study, we screened Aβ_42_ sequence- and conformation-specific antibodies 1F12 and 2C6 and selected preferred antibody pairs 1F12/2C6 and 1F12/1F12 for sandwich ELISA to accurately detect Aβ_42_Ms and Aβ_42_Os in blood and organs. Of note, in the results of identifying the epitopes of 1F12 and 2C6, we find that the 2C6 exhibited discrete epitopes. Presently, there are about five epitopes on the surface of Aβ_42_ peptides, mainly including Aβ_3__–__9_, Aβ_13__–__19_, Aβ_18__–__25_, Aβ_29__–__36_, and Aβ_36__–__42_ and several antibodies have been designed for the corresponding epitopes (De et al., 2019). Interestingly, our prepared 2C6 can recognize the Aβ_3__–__9_, Aβ_13__–__19_, Aβ_18__–__25_, Aβ_29__–__36_, but not for Aβ_36__–__42_. In the detailed analysis of the immune response of Aβ_36__–__42_, we used the keyhole limpet hemocyanin-coupled Aβ_36__–__42_ peptides to immunize BALB/c mice, but only one of three mice showed a weak immune response with a low titer. The inadequate immune response may be due to the Aβ_36__–__42_ peptides (VGGVVIA) containing many hydrophobic amino acids, such as V, I, and A, making it difficult for animals to absorb and followed to produce antibodies. Therefore, the explanation of why 2C6 exhibits discrete epitopes is that there are more hydrophilic or neutral amino acids instead of hydrophobic amino acids in the region of Aβ_1__–__36_, making it easier to be exposed and recognized by antibodies. Besides, the successful preparation of mAb A8978 (against the intermediate epitope Aβ_13__–__28_) and mAb 78 (against Aβ_7__–__11_, Aβ_18__–__24_, and Aβ_26__–__32_) indicate that it is possible to generate multiple epitope antibodies in the Aβ_1__–__36_ region (Hatami et al., 2014; Nabers et al., 2018), and all these results also further confirm our explanation. Studies describing the different roles of Aβ_42_ sequence region in the toxicity mechanisms have been reported by several groups. In general, the C-terminal area of Aβ_42_ plays a significant role in inducing bilayer permeability (De et al., 2019), while the middle and N-terminal parts of Aβ_42_ are effective in causing microglial inflammation and TLR signaling (Colvin et al., 2017; De et al., 2019). Therefore, antibodies targeting the C-terminal region of Aβ_42_ are more effective in reducing membrane permeability induced by Aβ_42_ aggregates, whereas antibodies targeting the middle and N-terminal areas of Aβ_42_ are more potent in reducing the inflammatory response induced by Aβ_42_ aggregates. These differences may be due to the accessibility of solvent-exposed N-terminal fragments in larger aggregates formed in the later stages of Aβ_42_ aggregation. Altogether, the functional effect of antibodies with more epitopes may be far greater than that of single-epitope antibodies, especially in terms of immunotherapy.

The 1F12/2C6 and 1F12/1F12 ELISAs provided sufficient sensitivity to detect the levels of Aβ_42_Ms and Aβ_42_Os in blood and tissue extracts of APP/PS1 mice, and the results were not affected by the peripheral expression of APP or Aβ_40_ because the prepared 1F12 and 2C6 showed more preference for Aβ_42_ species. The results of ELISA and immunoprecipitation (IP) performed in blood and brain extracts of 3- and 9-month-old APP/PS1 mice well confirmed that no APP protein brand was observed or detected, except for Aβ_42_Ms and several small Aβ_42_Os (Figures 1D, 2C,E, 3D,E). Longitudinal studies of blood Aβ_42_Ms and Aβ_42_Os levels showed extensive temporal variation within and among APP/PS1 mice that participated in our research. Based on the 1F12/2C6 and 1F12/1F12 ELISA tests, it was found that the decrease in the levels of total Aβ_42_ in blood was accompanied by its increase in the brain, but total Aβ_42_ levels in blood were not correlated with AD progression (*p* = 0.2146, Figure 3A). The results are unexpectedly consistent with the literature on Aβ_42_ detection because the Aβ_42_ level in previous reports did not show a significant change (Hoglund et al., 2005) using a pair of antibodies that recognize different Aβ_42_ epitopes, which is similar to 1F12/2C6 ELISA. In contrast, soluble Aβ_42_Os, whose levels are elevated in AD patients, are easily mis-detected in the measurement of Aβ_42_, resulting in underestimation of Aβ_42_Ms level and poor performance in assessing the progression of AD (Yang et al., 2013; Liu et al., 2017). However, based on our method, we found obvious decrease in the level of Aβ_42_Ms and significantly increased level of Aβ_42_Os in blood, and prominently elevated level of Aβ_42_Os in the brains of 3 and 9-month-old APP/PS1 mice (Figures 3B,C). Therefore, the levels of Aβ_42_Ms and Aβ_42_Os in blood and brain are closely associated with AD progression, which is helpful for us to further understanding of the pathogenesis of AD.

Increasing evidence indicates that the intestine or intestinal flora is associated with the progression of AD. However, to date, almost no literature directly reports the presence of Aβ_42_ in the intestinal tissues and further elaborates the relationship between Aβ_42_ in the intestine and AD progression. Our study showed that significantly increased levels of Aβ_42_ were observed in the gastrointestinal organs (including stomach, jejunum, ileum, colon, cecum, jejunum lysate, ileal lysate, and cecum lysate) of 9-month-old APP/PS1 mice but not in 3-month-old APP/PS1 mice or age-matched C57BL/6J mice (Figures 4, 5). The level of Aβ_42_Ms in the blood and brain of 3-month-old APP/PS1 mice was significantly higher than that in the intestine, indicating that the human Aβ produced by APP/PS1 mice is mainly distributed in the blood and brain. While the increasing Aβ_42_ levels in the gastrointestinal organs still needed more evidence to confirm whether it originates from the brain or/and blood. Presently, several potential mechanisms of how brain Aβ is released into peripheral tissues have been mentioned. Some convincing evidence suggests that brain-derived soluble Aβ_42_Ms and Aβ_42_Os can be absorbed by neurons and enter the intestine along the vagus nerve (Foley et al., 2020). In contrast, the administration of Aβ_42_ into the gastrointestinal tract may induce amyloidosis in the central nervous system (CNS) and AD-related pathologies, such as dementia (Sun et al., 2020). Besides, a study provided by Cintron et al. (2015) demonstrated the ability of peripheral monocytes to transport Aβ_42_ aggregates from the abdominal cavity to the brain, spleen, and liver. Several other groups claimed that Aβ seeds could be transported via axons (Clavaguera et al., 2014; Guo and Lee, 2014; Boluda et al., 2015; Ye et al., 2015). In addition, the levels of Aβ_42_ in the peripheral blood neuronal-derived exosomes in AD patients were higher than those in aMCI and healthy people, indicating that exosomes could also act as transport vehicles for Aβ_42_ (Jia et al., 2019; Lakshmi et al., 2020). Altogether, neurons, axons, neuronal-derived exosomes, and peripheral monocytes can be used as potential carriers of Aβ_42_ delivery, and there is currently evidence supporting axonal transport as a critical mode of disease propagation within the nervous system. In addition to the brain, other non-neural tissues, including the pancreas, kidney, spleen, heart, liver, testis, aorta, lung, skin, adrenal glands, and thyroid, also express amyloid-β protein precursor (APP). It is worth noting that the peripheral expression of APP will have the opportunity to be hydrolyzed to produce Aβ_42_ when APP is transferred to its resident site tans-Golgi network (TGN), where β- and γ-secretase are distributed (Roher et al., 2009; Querfurth and LaFerla, 2010; Sakono and Zako, 2010; Hodson, 2018). The produced Aβ_42_ is recycled to the cell membrane surface, which may increase the level of Aβ_42_ in peripheral tissues. However, to date, it is difficult to evaluate the impact of APP produced by peripheral tissues on Aβ levels in the brain and blood. The current evidence supports the brain as the primary source of Aβ pools because Aβ levels are the highest. Whether the source of APP or/and Aβ in peripheral tissues contributes to the Aβ pool of brain, blood or gastrointestinal organs and its influence on the detection of Aβ_42_ levels in the brain and blood need to be further confirmed to clarify.

In a detailed analysis of the intestinal origin of Aβ_42_, we found that Aβ_42_Ms and Aβ_42_Os were first detected in colonic lysates of 3-month-old APP/PS1 (Figures 4B–D, i). Interestingly, this phenomenon is partly consistent with the study reported by Hui et al. (2012) that the colon is considered the first segment of the gastrointestinal tract where Aβ deposits occur. This phenomenon may be explained that in the early stage of AD, the Aβ_42_, especially Aβ_42_ monomers or small oligomers from the brain and blood pools, can be easily transmitted through the enteric nervous system (ENS) to the digestive tract of the stomach and intestines. According to reports, the colon contains the most neurons (Gershon, 1998; Rao and Gershon, 2016; Brierley et al., 2018; Borgmann et al., 2021), and the monomers that gradually accumulate in the colon can be aggregated into small molecular weight oligomers. It is worth noting that the colon undergoes vital processes to ensure our health. These processes are coordinated by transmitting sensory signals from the periphery to the central nervous system, allowing communication from the intestine to the brain via the “gut-brain axis” (Brierley et al., 2018). Foley et al. (2020) have confirmed that soluble Aβ_42_Ms and Aβ_42_Os could be absorbed by neurons and enter the intestine along the vagus nerve. Therefore, there is a strong correlation between the colon and degenerative neurological disease. In general, our results and previous evidence may well explain the observation of Aβ_42_ in colon lysates, but the precise correlation of Aβ_42_ and the intestine still needs to be studied. Besides, the intestine and enteric nervous systems play an important role in neurological disorders and deserve our attention.

Compared to 3-month-old APP/PS1, we did not observe an increase in Aβ_42_ peptides in colonic lysates, although the levels of Aβ_42_Ms and Aβ_42_Os increased sharply in 9-month-old APP/PS1 (Figures 4B–D, iii). The significant difference in Aβ_42_ levels in colonic lysates of 3- and 9-month-old APP/PS1 mice may be due to the migration of Aβ_42_ in the colon from the colon to other gastrointestinal organs. This migration process has been confirmed by multiple groups (Cintron et al., 2015; Sun et al., 2020). In addition, high Aβ_42_ levels were detected in the cecum, ileum, jejunum, and even stomach of 9-month-old APP/PS1 mice. Among them, the Aβ_42_ level in the ileum was more significant and higher than that in the colon or colon lysate. These results further confirmed the ability of Aβ_42_ peptides to spread in the colon as seeds. Thus, the disappeared Aβ_42_ peptide in the colonic lysate of 9-month-old APP/PS1 may have migrated to other gastrointestinal organs such as the cecum, ileum, jejunum, and stomach because their Aβ_42_ levels were significantly higher than colon lysates. In summary, our study, together with previous studies, confirms the ability of Aβ_42_ peptides to spread as seeds, which leads to extensive changes in Aβ_42_ levels within and between different APP/PS1 mice.

In this study, we monitored the dynamic changes of Aβ_42_Ms and Aβ_42_Os levels in the intestines and studied their significance for AD. Indeed, our results convincingly demonstrated that Aβ exists in the intestines of APP/PS1 mice, and their levels are correlated with AD progression. However, a current controversy is whether the gastrointestinal organs can directly produce Aβ_42_. Several studies have reported that microorganisms including *Bacillus subtilis*, *Shigella*, *Escherichia coli*, *Salmonella enterica*, *Staphylococcus aureus*, and *Mycobacterium tuberculosis* can produce functional extracellular amyloid proteins but not for Aβ_42_ peptides (Hufnagel et al., 2013; Schwartz and Boles, 2013; Friedland, 2015; Pistollato et al., 2016). In fact, it seems that there is insufficient evidence to show that Aβ_42_ peptides could be directly produced in the organs of the gastrointestinal tract. In addition, the practical and accurate distinction between locally produced and transported Aβ_42_ peptides is crucial for helping to understand the pathology of AD. Distinguishing the local production and transportation of Aβ_42_ peptides is our next research plan.

In addition to finding elevated levels of soluble Aβ_42_, several insoluble Aβ_42_ plaques were observed in the intestines of 3-month-old APP/PS1 mice. However, it is in the early stages of AD, and there were only a few plaques in the brain (Figure 5, left). With the progression of AD, more plaques were observed in the intestines of 9-month-old APP/PS1 mice (Figure 5, right). Interestingly, changes in microbiome composition were observed in 9-month-old APP/PS1 mice. The effect of increased levels of Aβ_42_ in the intestine on the changes in its microbial composition remains obscure. There is no doubt that the observed toxicity of Aβ_42_, especially small oligomers, is the most effective in inducing inflammation of microglia or macrophages, and it causes more damage in inducing bilayer permeability (Flagmeier et al., 2017; Fusco et al., 2017; De et al., 2019). It is certain that in the current investigation, the abundance of Lachnospiraceae fluctuated with age (Figures 6C,D). The content of Turicibacter, Atopostipes, and Lachnospiraceae in aged APP/PS1 mice was significantly higher than in young APP/PS1 mice, but the abundance of Prevotellaceae in old APP/PS1 mice was lower (Figure 6). Overall, these results indicated that the significant difference in gut microbiota composition is accompanied by the sharply increased soluble Aβ_42_ and insoluble Aβ plaques during AD progression, but whether this change affects or is affected by the change in gut-derived Aβ_42_ needs to be further explored.

In summary, based on our screened antibody pairs 1F12/2C6 and 1F12/1F12 in sandwich ELISA for specific detection of Aβ_42_Ms and Aβ_42_Os, we observed apparent fluctuation in the levels of Aβ_42_Ms and Aβ_42_Os in blood and intestines of APP/PS1 mice during the progression of AD. The identification of Aβ_42_ abnormalities in the gastrointestinal tract could provide new ideas for AD therapeutic interventions that are only used to evaluate Aβ_42_ levels in peripheral blood and the brain. Furthermore, studying the relationship between the levels of Aβ_42_Ms and Aβ_42_Os in peripheral tissues and the progression of AD may help to understand the causes of AD, and may provide new treatment strategies for improving AD or other Aβ-related dementias.

## Data Availability Statement

The datasets presented in this study can be found in online repositories. The names of the repository/repositories and accession number(s) can be found in the article.

## Ethics Statement

The animal study was reviewed and approved by the Institutional Animal Care and Use Committee of Huazhong University of Science and Technology.

## Author Contributions

HL, QL, and ZZ coordinated the writing of the manuscript and provided writing guidance and manuscript revision. LZ completed all experiments and contributed to writing the first draft. YL and SN participated in most experiments. XL was involved in APP/PS1 mice breeding and schematic figure preparations. CY performed the bioinformatics data analysis. All authors reviewed and approved the final manuscript.

## Conflict of Interest

The authors declare that the research was conducted in the absence of any commercial or financial relationships that could be construed as a potential conflict of interest.

## Publisher’s Note

All claims expressed in this article are solely those of the authors and do not necessarily represent those of their affiliated organizations, or those of the publisher, the editors and the reviewers. Any product that may be evaluated in this article, or claim that may be made by its manufacturer, is not guaranteed or endorsed by the publisher.

## Supplementary Material

Supplementary Figure 1 Characterization of sequence- and conformation-specific antibodies 1F12 and 2C6 against Aβ_42_Ms and Aβ_42_Os. **(A)** The preferred hybridoma colonies were screened via indirect ELISA. **(B)** The purity and molecular weight of purified 1F12, 2C6, and 2E2 were confirmed via 12% reduced SDS-PAGE with Coomassie blue staining. **(C)** The bioactivities of 1F12, 2C6, and commercial antibody 6E10 toward natural Aβ_42_ peptides extracted from murine APP/PS1 brain were evaluated via Western blotting. **(D)** Confocal fluorescence images of murine APP/PS1 brain sections using Cy3-labeled anti-Aβ_42_ monoclonal antibody 2C6 and commercially available anti-Aβ antibody 6E10 **(E)**. (Scale bar: 200 μm). **(E)** Titers of 1F12, 2C6, and 2E2. **(F)** The purity and molecular weight of freshly prepared Aβ_42_Ms and Aβ_42_Os were determined by Western blotting with 2C6. **(G)** Binding affinities of 2C6 toward Aβ_42_Ms and Aβ_42_Os were evaluated via indirect ELISA. The activities **(H)** and titers **(I)** of the biotinylated 1F12 or 2C6 antibody.

Supplementary Figure 2 Confocal fluorescence images of whole murine APP/PS1 brain sections using Cy3-labeled anti-Aβ_42_ monoclonal antibody 1F12 **(A)** or 2C6 **(B)** and thioflavin S.

Supplementary Figure 3 Comparison of the dynamic distribution of Aβ_42_ in APP/PS1 and C57BL/6J. The levels of total Aβ_42_ **(A,D)**, Aβ_42_Os **(B,E)**, and Aβ_42_Ms **(C,F)** in the stomach, duodenum, jejunum, ileum, colon, and cecum and their lysates from APP/PS1 and C57BL/6J at 3-month-old (*n* = 4) or 9-month-old APP/PS1 (*n* = 4). Data are presented as means ± SEM. Two-way analysis of variance (ANOVA) was used for multigroup comparisons. Statistical significance is indicated in the figures by ***p* < 0.01, ****p* < 0.001 and *****p* < 0.0001.

Supplementary Figure 4 Representative confocal fluorescence images of the duodenum, jejunum, ileum, cecum, and colon of 3-month-old C57BL/6J mice double-stained with thioflavin S and Cy3-1F12 (Scale bar: 100 μm).

Supplementary Figure 5 Representative confocal fluorescence images of the duodenum, jejunum, ileum, cecum, and colon of 9-month-old C57BL/6J mice double-stained with thioflavin S and Cy3-1F12 (Scale bar: 100 μm).
