# Supplementary material for: Dynamic Changes in the Levels of Amyloid-β42 Species in the Brain and Periphery of APP/PS1 Mice and Their Significance for Alzheimer’s Disease
Source: Front Mol Neurosci. 2021 Aug 27;14:723317. doi: 10.3389/fnmol.2021.723317 (PMC8430227; doi:10.3389/fnmol.2021.723317)
Supplement: Supplementary Figure 1 — Characterization of sequence- and conformation-specific antibodies 1F12 and 2C6 against Aβ42Ms and Aβ42Os. (A) The preferred hybridoma colonies were screened via indirect ELISA. (B) The purity and molecular weight of purified 1F12, 2C6, and 2E2 were confirmed via 12% reduced SDS-PAGE with Coomassie blue staining. (C) The bioactivities of 1F12, 2C6, and commercial antibody 6E10 toward natural Aβ42 peptides extracted from murine APP/PS1 brain were evaluated via Western blotting. (D) Confocal fluorescence images of murine APP/PS1 brain sections using Cy3-labeled anti-Aβ42 monoclonal antibody 2C6 and commercially available anti-Aβ antibody 6E10 (E). (Scale bar: 200 μm). (E) Titers of 1F12, 2C6, and 2E2. (F) The purity and molecular weight of freshly prepared Aβ42Ms and Aβ42Os were determined by Western blotting with 2C6. (G) Binding affinities of 2C6 toward Aβ42Ms and Aβ42Os were evaluated via indirect ELISA. The activities (H) and titers (I) of the biotinylated 1F12 or 2C6 antibody. [file Image_1.pdf]

Dynamic changes in the levels of amyloid-β_{42} species in the brain and periphery of APP/PS1 mice and their significance for Alzheimer's disease

Liding Zhang[1,2], Changwen Yang[1,2], Yanqing Li[1,2], Shiqi Niu[1,2], Xiaohan Liang[1,2], Zhihong Zhang[1,2,3], Qingming Luo[1,2,3], Haiming Luo[1,2*]

[1]Britton Chance Center for Biomedical Photonics, Wuhan National Laboratory for Optoelectronics-Huazhong University of Science and Technology, Wuhan, China

[2]MoE Key Laboratory for Biomedical Photonics, School of Engineering Sciences, Huazhong University of Science and Technology, Wuhan, China

[3]School of Biomedical Engineering, Hainan University, Haikou, Hainan 570228, China

***Correspondence:** Haiming Luo, hemluo@hust.edu.cn;

Britton Chance Center for Biomedical Photonics, Wuhan National Laboratory for Optoelectronics–Huazhong University of Science and Technology, 430074, Wuhan, Hubei, China. Fax: +86-27-87792034; Tel: +86-27-87792033;

Supplementary Material

Table of Contents

Supplementary Figure 1. Characterization of sequence- and conformation-specific antibodies 1F12 and 2C6 against $A\beta_{42}$Ms and $A\beta_{42}$Os.

Supplementary Figure 2. Confocal fluorescence images of murine APP/PS1 brain sections using Cy3-labeled anti-$A\beta_{42}$ monoclonal antibody 1F12 or 2C6 and thioflavin S.

Supplementary Figure 3. Comparison of the dynamic distribution of $A\beta_{42}$ in APP/PS1 (n = 4) and C57BL/6J (n = 4).

Supplementary Figure 4. Representative confocal fluorescence images of the duodenum, jejunum, ileum, caecum, and colon of 3-month-old C57BL/6J mice double-stained with thioflavin S and Cy3-1F12.

Supplementary Figure 5. Representative confocal fluorescence images of the duodenum, jejunum, ileum, caecum, and colon of 9-month-old C57BL/6J mice double-stained with thioflavin S and Cy3-1F12.

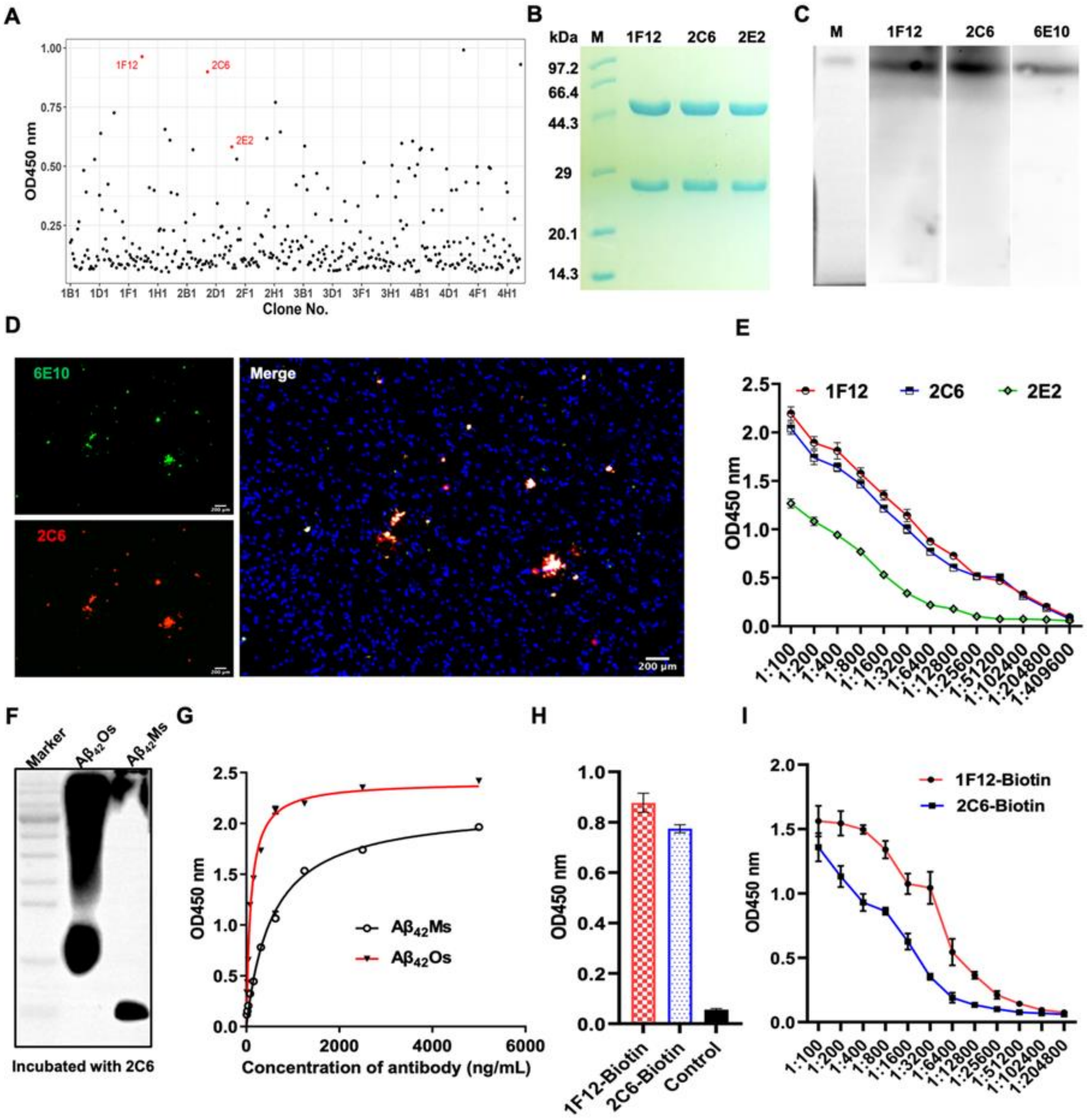

Supplementary Figure 1 Characterization of sequence- and conformation-specific antibodies 1F12 and 2C6 against Aβ_{42}Ms and Aβ_{42}Os. **A**, The preferred hybridoma colonies were screened via indirect ELISA. **B**, The purity and molecular weight of purified 1F12, 2C6 and 2E2 were confirmed via 12% reduced SDS-PAGE with Coomassie blue staining. **C**, The bioactivities of 1F12, 2C6, and commercial antibody 6E10 toward natural Aβ_{42} peptides extracted from murine APP/PS1 brain were evaluated via Western blotting. **D**, Confocal fluorescence images of murine APP/PS1 brain sections using Cy3-labeled anti-Aβ_{42} monoclonal antibody 2C6 and commercially available anti-Aβ antibody 6E10. (Scale bar: 200 μm). **E**, Titers of 1F12, 2C6, and 2E2. **F**, The purity and molecular weight of freshly prepared Aβ_{42}Ms and Aβ_{42}Os were determined by Western blotting with 2C6. **G**, Binding

affinities of 2C6 toward Aβ_{42}Ms and Aβ_{42}Os were evaluated via indirect ELISA. The activities (**H**) and titers (**I**) of the biotinylated 1F12 or 2C6 antibody.

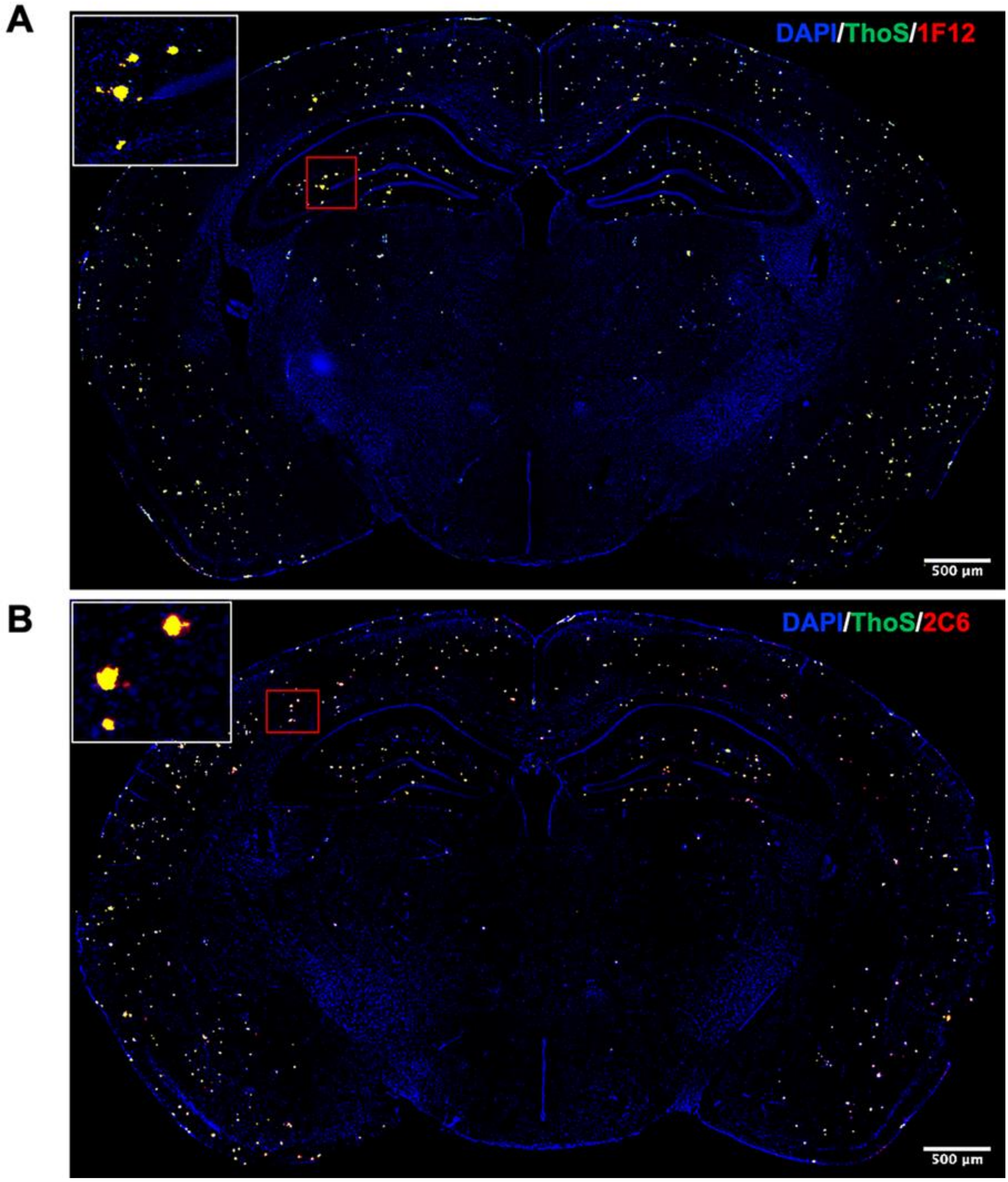

Supplementary Figure 2. Confocal fluorescence images of whole murine APP/PS1 brain sections using Cy3-labeled anti-Aβ_{42} monoclonal antibody 1F12 (**A**) or 2C6 (**B**) and thioflavin S.

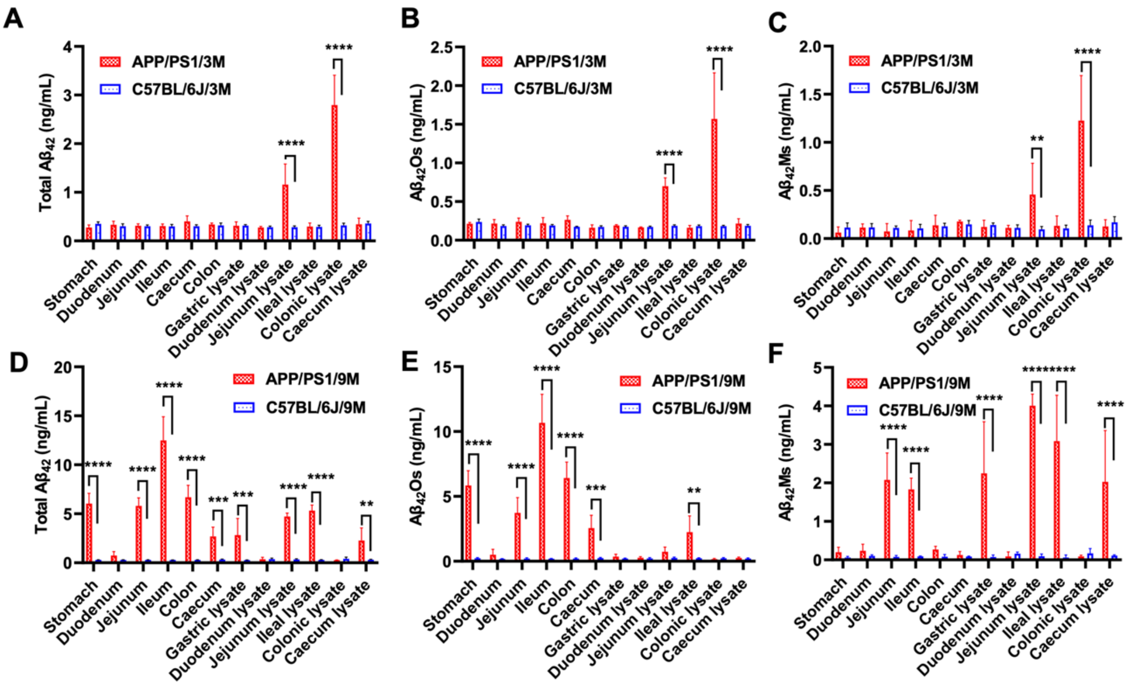

Supplementary Figure 3. Comparison of the dynamic distribution of Aβ42 in APP/PS1 and C57BL/6J. The levels of total Aβ42 (**A, D**), Aβ42Os (**B, E**), and Aβ42Ms (**C, F**) in the stomach, duodenum, jejunum, ileum, colon, and caecum and their lysates from APP/PS1 and C57BL/6J at 3-month-old (n = 4) or 9-month-old APP/PS1 (n = 4). Data are presented as means ± SEM. Two-way analysis of variance (ANOVA) was used for multigroup comparisons. Statistical significance is indicated in the figures by $^{**}p < 0.01$, $^{***}p < 0.001$ and $^{****}p < 0.0001$.

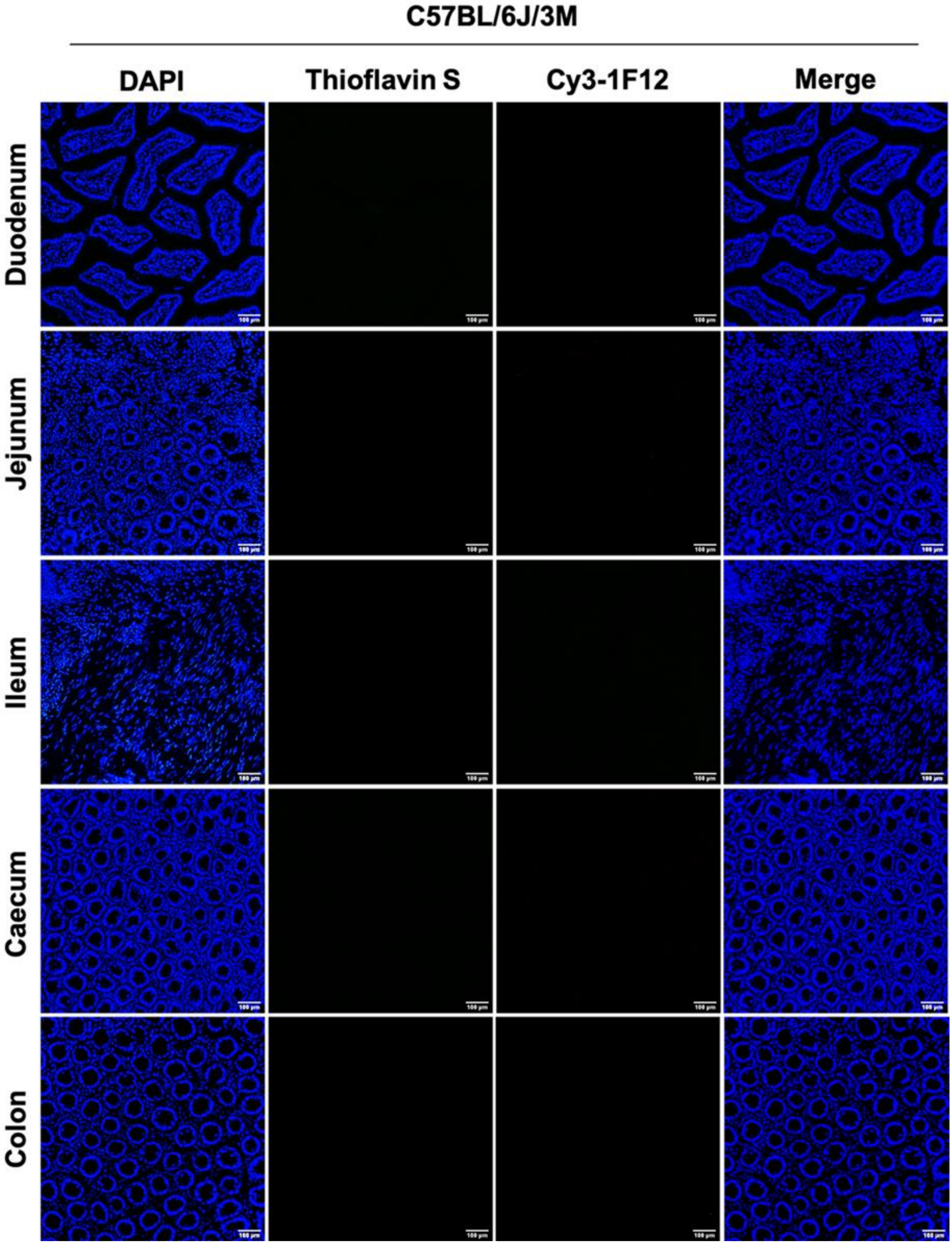

Supplementary Figure 4. Representative confocal fluorescence images of the duodenum, jejunum, ileum, caecum, and colon of 3-month-old C57BL/6J mice double-stained with thioflavin S and Cy3-1F12, (Scale bar: 100 μm).

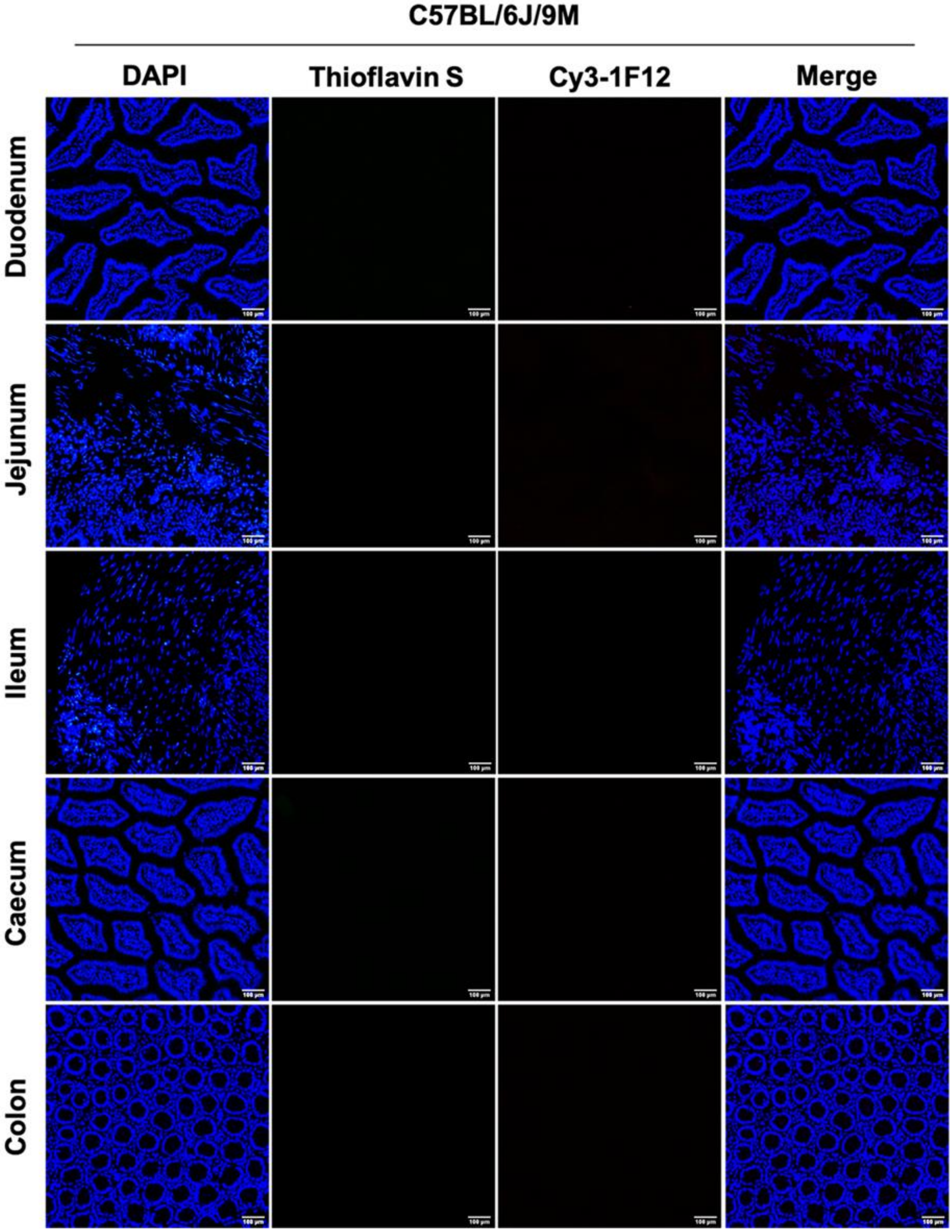

Supplementary Figure 5. Representative confocal fluorescence images of the duodenum, jejunum, ileum, caecum, and colon of 9-month-old C57BL/6J mice double-stained with thioflavin S and Cy3-1F12, (Scale bar: 100 μm).
